# Nasal microbes in allergic rhinitis children with or without sublingual immunotherapy

**DOI:** 10.1097/MD.0000000000035711

**Published:** 2023-10-27

**Authors:** Xiao-Fei Shen, Zhi-Pan Teng, Qi Li, Zhen-Kun Yu

**Affiliations:** a Department of Otorhinolaryngology, Children’s Hospital of Nanjing Medical University, Nanjing, Jiangsu Province, China; b Department of Otorhinolaryngology, BenQ Medical Center, The Affiliated BenQ Hospital of Nanjing Medical University, Nanjing, Jiangsu Province, China.

**Keywords:** allergic rhinitis, children, nasal microbes, sublingual immunotherapy

## Abstract

The mechanism of allergic rhinitis (AR) remains unclear. Most researchers believe that AR is the result of a combination of environmental and genetic factors. Sublingual immunotherapy (SLIT) is a treatment that can change the natural course of AR through immunomodulatory mechanism and maintain efficacy after the treatment. Nasal cavity is the main site where AR patients contact with external allergens, produce inflammatory reactions and nasal symptoms. Therefore, in this study, we investigate the nasal microbiome in AR patients, and the changes after SLIT. In this cross-sectional study, nasal swabs for microbiome analysis were collected from 3 groups: SLIT-naïve AR patients (AR group), AR patients undergoing SLIT treatment over 2 years (SLIT group) and a control group (CG). The characteristics of nasal microbiome of each groups were produced by 16s-rDNA sequencing technology. The Simpson index of AR group was significantly higher than that of CG and SLIT groups, but not different between SLIT group and CG group. The abundance of *Bacteroidete* and *Firmicutes* remarkably increased in the AR group, but *Bacteroidete* reduced to CG level after SLIT. AR patients have different nasal microbiome composition, but we do not know how it happened and whether the AR condition affected nasal microbiome composition or nasal microbiome affected AR.

## 1. Introduction

Allergic rhinitis (AR) is an immunoglobulin E (IgE)-mediated inflammatory response to inhaled particles in nasal cavity.^[1]^ Epidemiological studies have revealed that the prevalence of AR has increased progressively in developed countries, and affects 10% to 40% of adults and 2% to 25% of children worldwide.^[2]^ It is also a critical public health, medical and economic problem in China, the prevalence of AR in the 18 major cities is17.6%, which increased gradually, especially in children.^[3]^

The mechanism of AR is still unclear. Most scholars believe that AR is the result of the interactive effect of environmental and genetic factors. As the “health hypothesis” said, opportunities for children to be exposed to pathogenic microorganisms in the natural environment were significantly reduced as the human living environment improved, therefore the immune system cannot receive an effective stimulus and schedules,^[4]^ which caused the autoimmune system overreacted to the normal flora of the body, thus increased the prevalence of allergic diseases year by year.

Current evidences demonstrated that 3 to 5 years of allergen immunotherapy (AIT), which could cause allergen specific immune system tolerance, thereby reduced the nasal symptoms and blocked the natural processes of allergic diseases.^[5,6]^ Both subcutaneous immunotherapy (SCIT) and sublingual immunotherapy (SLIT) have been proved to be effective and safe by previous studies.^[7]^ SLIT is more acceptable for children since it has fewer and less severe adverse events compared with SCIT.^[8]^ AIT is applied to clinical practices for almost a century, clinical efficacy has also been widely recognized, but its specific mechanism is still unclear.

There were few researches about the relationships between nasal microbiome dysbiosis and the developments of AR, whereas the nasal microbiome holds an important role in the modulation of localized immune responses, pathophysiology, and development of AR potentially.^[9]^ Nasal cavity is the main place where AR children contact with external allergens, produce inflammatory reactions and nasal symptoms. What is its microbial community composition? Are there any differences between healthy controls or AR children after SLIT? Is the mechanism of SLIT related to the microbes in the nasal cavity? Further research is needed to fully comprehend the role of nasal microbiome dysbiosis in AR.

Therefore, in this study, we take the nasal microbiome as an entry point to compare the nasal microbiome differences between the healthy controls, AR children before and after SLIT.

## 2. Materials and methods

### 2.1. Patients and sample

This study was approved by the Ethics Committee of Children’s Hospital of Nanjing Medical University. Informed consents were obtained from the guardians of participants. A total of 45 children were randomly enrolled from the Department of Otorhinolaryngology, Children’s Hospital of Nanjing Medical University from July 2022 to August 2022. Include: Fifteen AR patients undergoing SLIT over 2 years (SLIT group); Fifteen SLIT-naïve AR patients (AR group); A control group with 15 heathy children (control group [CG]). The AR patients were confirmed by Guideline for diagnosis and treatments of pediatric allergic rhinitis (2022, revision).^[10]^ All AR patients received allergen skin prick test and/or allergen serum-specific IgE test, and the positive results included at least 1 mite allergen. Exclusion criteria were: rhinosinusitis with or without polyposis, cystic fibrosis, autoimmune conditions affecting nasal mucosa, sinonasal tumor, aspirin-exacerbated respiratory disease, allergic fungal rhinosinusitis, prior history of sinus surgery, and treatment with systemic or topical antibiotics or glucocorticoid in the preceding 4 weeks.

The age and gender of all children and the total nasal symptom score (TNSS) of AR were recorded. Samples of each children were collected from the common meatus under anterior rhinoscopy. The nasal swabs were inserted into each nostril, rotated for 3 times, applied constant pressure and placed immediately into a sterile tube. Samples were immediately transported to the microbiology laboratory and kept at −80°C for further analysis.

### 2.2. 16S-rDNA sequencing

DNA from samples was extracted by CTAB according to manufacturer’s instructions. The reagent which was designed to uncover DNA from trace amounts of sample has been proved to be effective for the preparation of DNA of most bacteria. Nuclear-free water was used for blank. The total DNA was eluted in 50 μL of elution buffer and stored at −80 °C until measurement in the PCR by LC-Bio Technology Co., Ltd, Hang Zhou, Zhejiang Province, China.

The primers: 341F(5’-CCTACGGGNGGCWGCAG-3’) and 805R(5’-ACTACHVGGGTATCTAATCC-3’)^[11]^ were tagged with specific barcodes per sample and sequenced universal primers. PCR amplification was performed in a total volume of 25 μL reaction mixture containing 25 ng of template DNA, 12.5 μL PCR Premix, 2.5 μL of each primer, and PCR-grade water to adjust the volume. The PCR conditions to amplify the prokaryotic 16S fragments consist of an initial denaturation at 98 °C for 30 seconds, 32cycles of denaturation at 98 °C for 10 seconds, annealing at 54°C for 30 seconds, and extension at 72 °C for 45 seconds; and then final extension at 72 °C for 10 minutes. The PCR products were confirmed with 2% agarose gel electrophoresis. Throughout the DNA extraction process, ultrapure water, instead of a sample solution, was used to exclude the possibility of false-positive PCR results as a negative control. The PCR products were purified by AMPure XT beads (Beckman Coulter Genomics, Danvers, MA) and quantified by Qubit (Invitrogen, USA). The amplicon pools were prepared for sequencing the size, and quantity of the amplicon library were assessed on Agilent 2100 Bioanalyzer (Agilent, USA) and with the Library Quantification Kit for Illumina (Kapa Biosciences, Woburn, MA), respectively. The libraries were sequenced on NovaSeq PE250 platform.

### 2.3. Sequencing data processing

Samples were sequenced on an Illumina NovaSeq platform according to the manufacturer’s recommendations, provided by LC-Bio. Paired-end reads was assigned to samples based on their unique barcode and truncated by cutting off the barcode and primer sequence. Paired-end reads were merged using FLASH. Quality filtering on the raw reads were performed under specific filtering conditions to obtain the high-quality clean tags according to the fqtrim (v0.94). Chimeric sequences were filtered using Vsearch software (v2.3.4). After dereplication using DADA2, we obtained amplicon sequence variants feature table and amplicon sequence variants feature sequence.

### 2.4. Data analysis

SPSS 19.0 software was used for statistical analysis of the general data of patients. Age and the TNSS among groups were tested by *T* test and gender comparison among groups was compared by Chi-square test. *P *< .05 was considered as statistically significant. Bioinformation sequence were mainly analyzed using the QIIME2 and the diagrams were implemented using the R package (v3.5.2).

QIME2 software was preferred to draw dilution curves for saturation analysis to evaluate whether the current sequencing depth was sufficient to include microbial diversity. Alpha diversity is applied to analyze complexity of species diversity for a sample through 5 indices, including Chao1, Observed-otus, Shannon, Simpson, and Goods-coverage. The nonmetric multidimensional scaling based on weighted UniFrac distances of the samples was used for beta diversity analysis by QIIME2 and R package

Then according to SILVA (release 138)^[12]^ classifier, feature abundance was normalized using relative abundance of each sample. The wilcox test was used to compare the differences between the 2 groups of samples with biological replicates, and the kruskal–Wallis test was used to compare multiple groups with biologically replicated samples. LDA effect size (LEfSe) analysis was used to find species with significant abundance differences among different groups (biomarkers). Blast was used for sequence alignment, and the feature sequences were annotated with SILVA database for each representative sequence.

## 3. Results

### 3.1. Population characteristics and TNSS

A total of 45 pediatric subjects were enrolled,15 children in each group. There were 5 female and 10 male in the CG (mean age = 6.75 ± 2.12), 7 female and 8 male in AR group (mean age = 7.41 ± 1.58), and 5 female and 10 male in SLIT group (mean age = 8.01 ± 1.43). The mean ages and gender among the 3 groups have no statistically difference (*P* > .05). The TNSS of the AR patients in the SLIT (TNSS = 4.47 ± 2.696) were significantly lower than the AR group (TNSS = 7.07 ± 2.086) (*P* = .006), which confirmed the efficacy of SLIT treatment.

### 3.2. Alpha diversity

**Figure 1.**
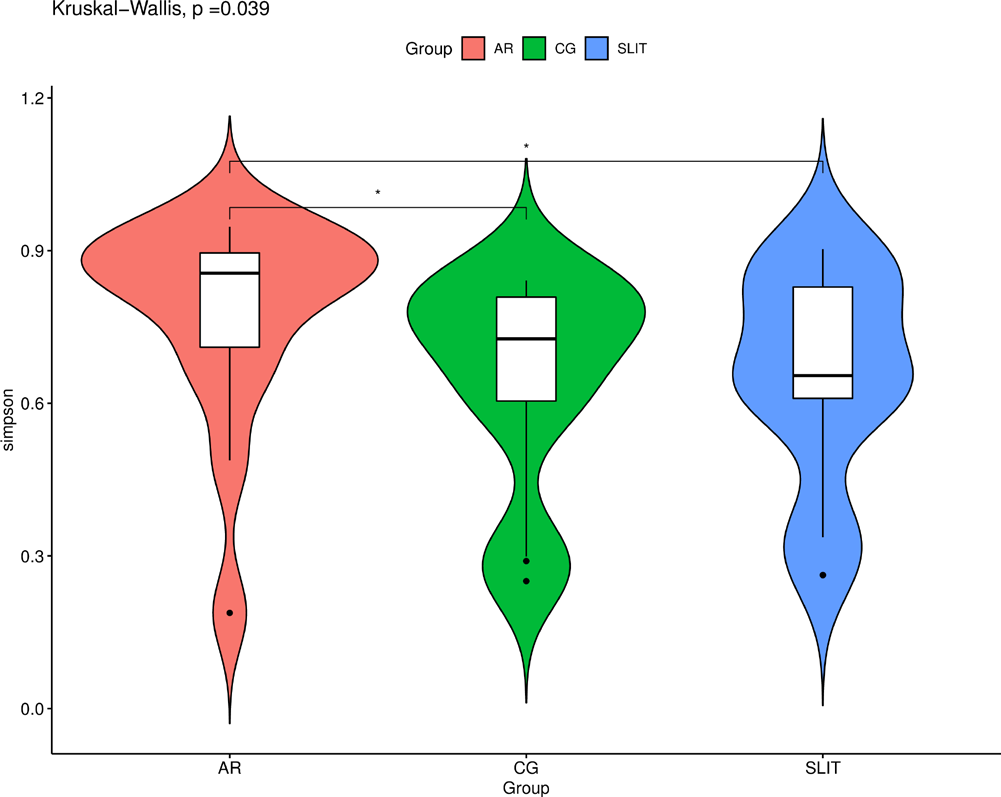
Violin plot of simpson index in alpha diversity. AR: SLIT-naïve AR patients, SLIT: AR patients undergoing SLIT treatment over 2 years. There are significant differences between the groups marked with * (*P < *.05). AR = allergic rhinitis, CG= control group, SLIT = sublingual immunotherapy.

Kruskal–Wallis test revealed that there was no significant difference in chao1, observed-otus and shannon indices among the 3 groups (Table 1). There was a statistically significant difference of simpson index among 3 groups (*P* = .039), the simpson index had significantly increased in the AR group compared to the control group (*P = .02*1) and the SLIT group (*P = .045*), but there is no difference between the control group and SLIT group (Fig. 1).

**Table 1:** The analysis of alpha diversity.

Alpha_diversity	CG	AR	SLIT	*P* value
Shannon	2.79 ± 0.99	3.83 ± 1.49	2.87 ± 1.07	#=.065
Simpson	0.65 ± 0.21	0.78 ± 0.21	0.66 ± 0.21	#=.039, *=.021, ^=.045,/=0.74
Observed-otus	176.93 ± 37.76	217.07 ± 65.53	214 ± 55.95	#=.096
Chao1	182.24 ± 40.54	221.27 ± 64.52	218.93 ± 58.15	#=.095

AR group: SLIT-naïve AR patients, SLIT group: AR patients undergoing SLIT treatment over 2 years. #: CG vs AR vs SLIT, *: CG vs AR, ^: AR vs SLIT, /: CG vs SLIT, *P *< .05 was considered as statistically significant.

AR = allergic rhinitis, CG = control group, SLIT = sublingual immunotherapy.

### 3.3. Beta diversity

**Figure 2.**
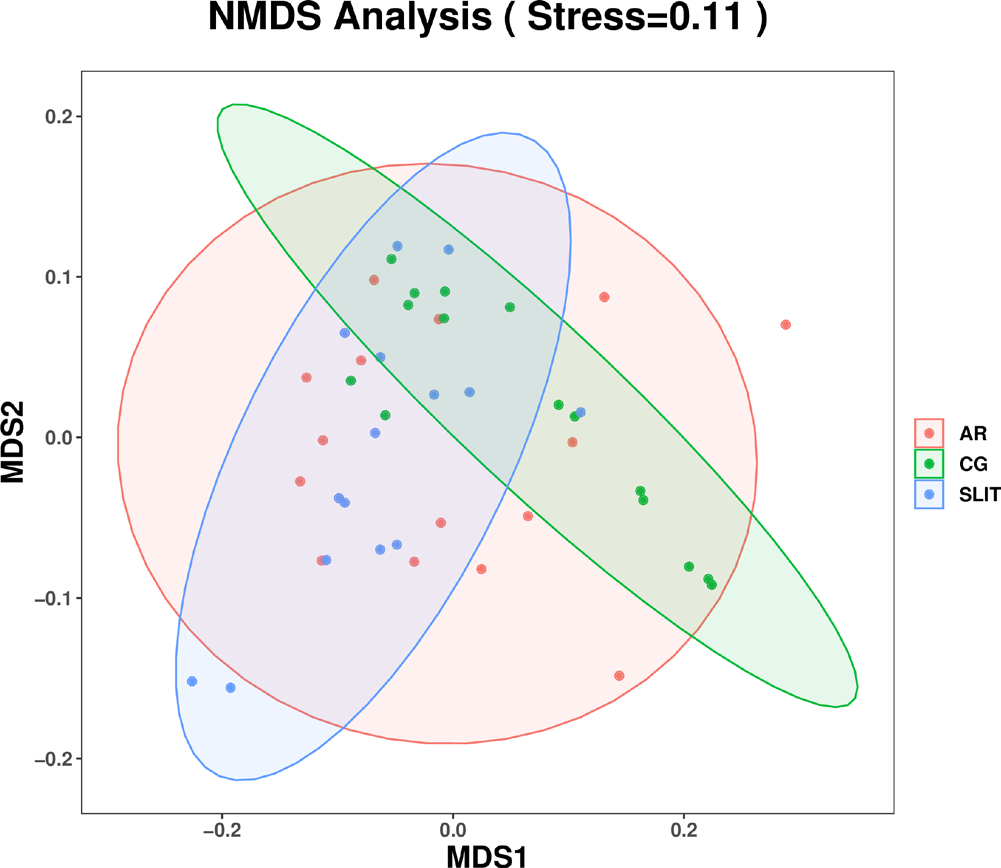
The weighted_unifrac_NMDS plot of bete diversity. Note: AR: SLIT-naïve AR patients, SLIT: AR patients undergoing SLIT treatment over 2 years. The points in the figure represent samples, and samples of different colors belong to different groups. The distance between points represents the degree of difference between samples. The stress coefficient is used to measure the quality of NMDS analysis results. Stress < 0.2 means the NMDS plot has certain explanatory significance, stress < 0.1 considered as a good sorting; stress < 0.05 is very representative. AR = allergic rhinitis, CG = control group, NMDS = nonmetric multidimensional scaling, SLIT = sublingual immunotherapy.

Beta diversity analysis focused on reflecting the similarity of community structure among different samples. Nonmetric multidimensional scaling analysis based on weighted UniFrac distance found no obvious clustering among groups (Fig. 2).

### 3.4. Species analysis

**Figure 3.**
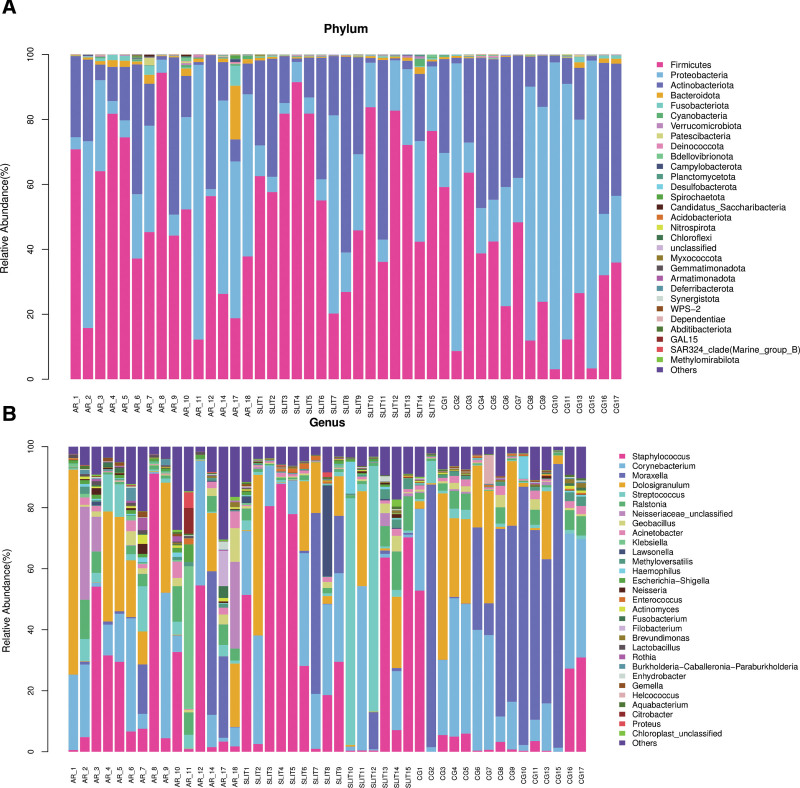
The stacked bar chart of top 30 species in relative abundance (%) of each sample at phylum (A) and genus (B) level. AR: SLIT-naïve AR patients, SLIT: AR patients undergoing SLIT treatment over 2 years. The horizontal axis represents the sample name, and the vertical axis represents the relative abundance of a certain classification. Different colors correspond to different species at the same level. AR = allergic rhinitis, CG = control group, SLIT = sublingual immunotherapy.

**Figure 4.**
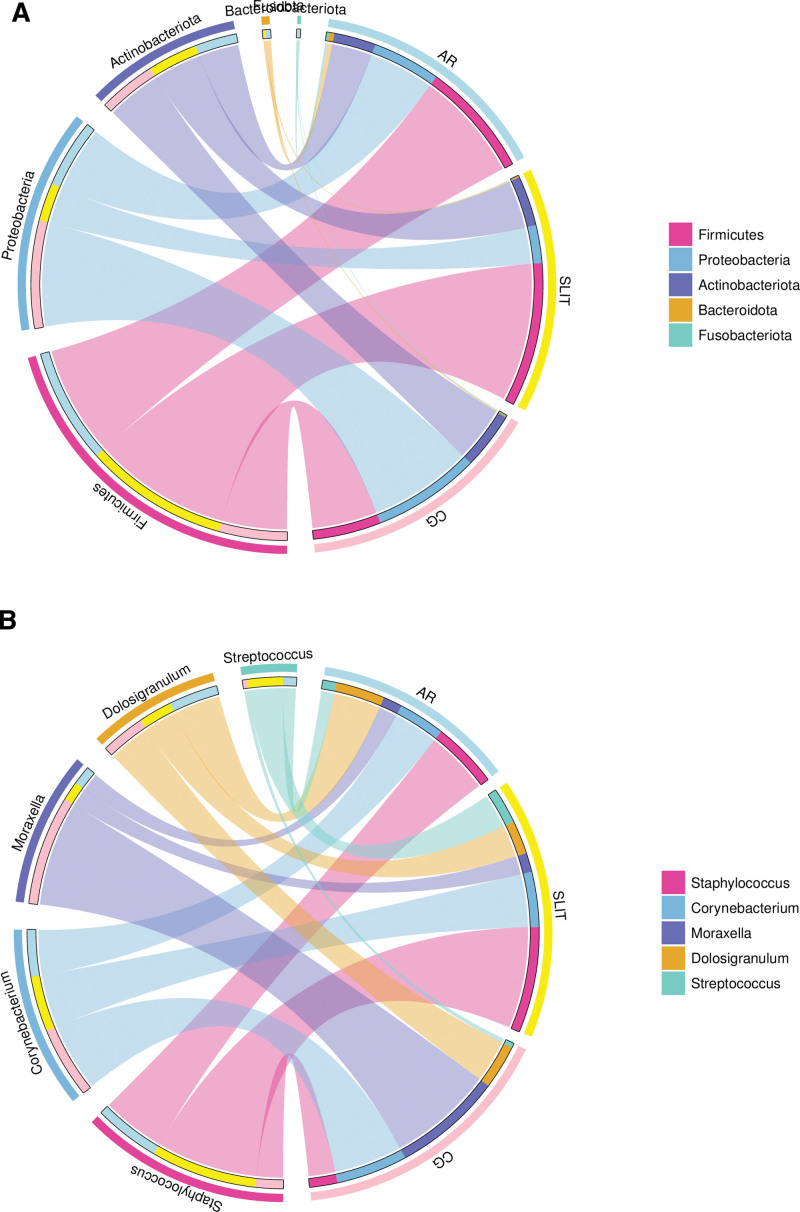
The distribution of the top 5 species in Circos diagram in each group at phylum (A) and genus (B) level. AR: SLIT-naïve AR patients, SLIT: AR patients undergoing SLIT treatment over 2 years. AR = allergic rhinitis, CG = control group, SLIT = sublingual immunotherapy.

The top 30 species in relative abundance among all samples had been shown at phylum (Fig. 3A) and genus level (Fig. 3B). Top 5 species relative abundance of nasal cavity included *Firmicutes, Proteobacteria, Actinobacteriota, Bacteroidota*, and *Fusobacteriota* at the phylum level (Fig. 4A), and *Staphylococcus, Corynebacterium, Moraxella, Dolosigranulum*, and *Streptococcus* at the genus level (Fig. 4B). At phylum, the richness of *Firmicutes* (CG:28.3%, AR:48.7%, SLIT:61.13%) were significant higher in AR group and SLIT group than CG group, and *Bacteroidota* (CG:0.50%, AR:2.18%, SLIT:0.57%) was significantly higher in AR group, but reduced to CG level in the SLIT group (Table 2). At genus level (Table 3), the *Staphylococcus* (CG:0.50%, AR:21.67%, SLIT:34.63%) in AR and SLIT group had higher relative abundance than the CG group, but *Moraxella* (CG:35.81%, AR:6.23%, SLIT:6.27%) in the AR and SLIT group were significantly lower than CG group.

**Table 2:** The analysis of the top 5 species in relative abundance (%) of at the phylum level.

Phylum	Control	AR	SLIT	*P* value
Firmicutes	28.83	48.79	61.13	#=.00, *=.03, ^=.13, /=.00
Proteobacteria	45.65	28.96	16.14	#=.03, *=.10, ^=.44, /=.01
Actinobacteriota	24.22	17.81	21.21	#=.57, *=.33, ^=.72, /=.44
Bacteroidota	0.50	2.18	0.57	#=.01, *=.01, ^=.03, /=.24
Fusobacteriota	0.32	0.94	0.16	#=.54, *=.31, ^=.37, /=.92

AR group: SLIT-naïve AR patients, SLIT group: AR patients undergoing SLIT treatment over 2 years. #: CG vs AR vs SLIT, *: CG vs AR, ^: AR vs SLIT,/: CG vs SLIT, *P *< .05 was considered as statistically significant.

AR = allergic rhinitis, CG = control group, SLIT = sublingual immunotherapy.

**Table 3:** The analysis of relative abundance (%) of species at the genus level.

Genus	Control	AR	SLIT	*P* value
Staphylococcus	9.11	21.67	34.63	#=.04, *=.04, ^=.63, /=.02
Corynebacterium	23.37	15.35	18.11	#=.27, *=.14, ^=.72, /=.21
Moraxella	35.81	6.23	6.27	#=.08, *=.04, ^=.98, /=.06
Dolosigranulum	14.46	16.05	10.95	#=.67, *=.63, ^=.82, /=.33
Streptococcus	1.45	4.40	11.69	#=.13, *=.04, ^=.19, /=.76

AR group: SLIT-naïve AR patients, SLIT group: AR patients undergoing SLIT treatment over 2 years. #: CG vs AR vs SLIT, *: CG vs AR, ^: AR vs SLIT, /: CG vs SLIT, *P *< .05 was considered as statistically significant.

AR = allergic rhinitis, CG = control group, SLIT = sublingual immunotherapy.

**Figure 5.**
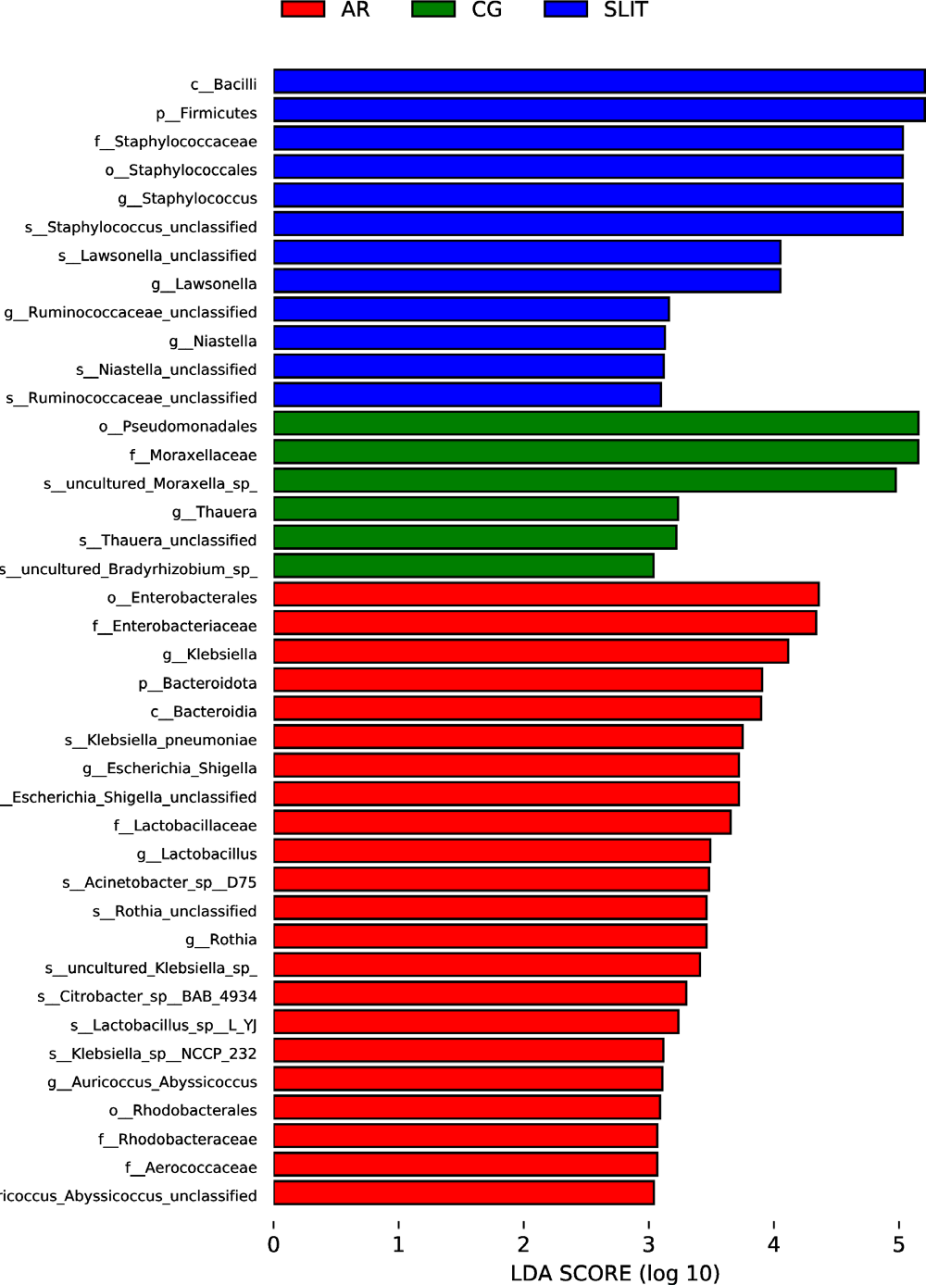
The bar graph of biomakers of LDA Effect Size analysis in each group. AR: SLIT-naïve AR patients, SLIT: AR patients undergoing SLIT treatment over 2 years. The color of the bar graph represents the group in which each species is more abundant, while the length represents the LDA score, which is the degree of influence of biomaker between different groups. AR = allergic rhinitis, CG = control group, SLIT = sublingual immunotherapy.

LEfSe analysis was used to find the biomarkers of the nasal cavity in each group (Fig. 5). Focus on the phylum level, the biomarkers was *Bacteroidota* in the AR group but *Firmicutes* in the SLIT group. At the genus level, the biomarkers included Klebsiella, Escherichia, Lactobacillus, Rothia and Auricoccus in the AR group, *Staphylococcus, Lawsonella, Niastella*, and *Ruminococcaceae-unclassified* in the SLIT group and *Thauera* in the CG group.

## 4. Discussion

The human microbiome consists of the collective genomes of commensal, symbiotic and pathogenic. LEfSe analysis was used to find species with significant abundance differences among different groups (biomarkers)living on the human body and playing a key role in health and immunity. Each body habitat harbors a characteristic bacterial community.^[13]^ Dysbiosis, a dysfunction or imbalance in the microbial communities, can therefore greatly impact human health.^[14]^ In recent years, bacterial communities of the nasal cavity had been revealed to be involved in the development of AR.^[15]^ The rapid increase in the prevalence of AR in children has attracted global attention. Epidemiological studies showed that the diagnosed rate of AR in children was 10.80% to 21.09%, with an increasing trend in China.^[16]^ Although most patients achieve beneficial effects from medicine therapy, AIT is the only treatment that can alter the natural course of AR disease through immunomodulatory mechanisms and maintain the efficacy after the course.^[17]^ Our study found significant differences in alpha diversity and microbial composition among the AR group, SLIT group and CG group.

The α-diversity across samples shown a statistically significant difference of simpson index among 3 groups, but no difference in Chao1 and Observed and shannon indices. These demonstrated that there was on significant group difference in richness but did show a difference in evenness, consistent with previous research reports.^[18]^ We make a pairwise further comparison of the 3 groups, and revealed that the simpson index of AR group was significantly higher than the CG group and the SLIT group, but there is no difference between the CG group and SLIT group. These shown that the bacterial evenness of AR patients had increased in their nasal microbiomes, as prior studies reports,^[19,20]^ but had an apparently fallen after 2 years SLIT treatment.

About the composition of microbes, we found a significant increase of the abundance of *Firmicutes* and *Bacteroidetes* in AR group compared to the CG group. Studies on intestinal microbes in adult asthma patients found that the relative abundance of *Firmicutes* and *Bacteroidetes* in asthma patients increased, which was significantly associated with the severity of allergy and lung function.^[21]^ Thus, we could infer that the plus of *Firmicutes* and *Bacteroidetes* in nasal cavity might be also related to the mechanism of AR. *Bacteroidetes* as the biomarker of nasal cavity in the AR patients based on the LEfSe analysis deserved a special attention. In our study, the richness of *Bacteroidetes* was significantly increased in the AR patients, but reduced after SLIT, which may be related to the mechanism of SLIT in AR. In addition, the significantly increased of *Staphylococcus* genus in AR group compared to control group had been reported in a national study yet.^[22]^ We also found a remarkably increase of *Staphylococcus* (member of *Firmicutes*) in AR patients. All these variations in nasal microbiota may contribute to the appearance and development of AR.

Although our study focusing on children greatly reduced the effects of demographic variables on the alterations in the nasal microbiome, such as smoking or age.^[23]^ There are still some limitations to this study. First of all, this was just a small sample size cross-sectional study captured on 1 time point, a large sample longitudinal follow-up of AR patients undergoing SLIT treatment is needed, which allows for serial repeated measures of the nasal microbiome status. Then, our study can learn the different microbiota between the AR group and the control group, or between the AR group and the SCIT group, but we do not know the different microbiota has a positive or negative regulatory effect on AR inflammation or how does it happen. Therefore, further animal model studies and clinical correlation studies are needed.

## 5. Conclusion

This study evaluated the changes about the bacterial nasal microbiome what occurred in AR children before and after SLIT. The bacterial evenness of nasal microbiome increased in the AR patients and reduced in the patients undergoing SLIT over 2 years. The abundance of *Firmicutes* and *Bacteroidete* were remarkably increased in the AR patients, and the *Bacteroidete* reduced in the patients undergoing SLIT over 2 years. All these variations in nasal microbiota may contribute to the mechanism of AR and SLIT.

## Author contributions

**Data curation:** Zhi-Pan Teng.

**Investigation:** Xiao-Fei Shen, Zhenkun Yu.

**Methodology:** Xiao-Fei Shen, Zhi-Pan Teng.

**Writing – original draft:** Zhi-Pan Teng.

**Writing – review & editing:** Xiao-Fei Shen, Qi Li, Zhenkun Yu.
